# Acceptance as an Emotion Regulation Strategy in Experimental Psychological Research: What We Know and How We Can Improve That Knowledge

**DOI:** 10.3389/fpsyg.2020.00242

**Published:** 2020-02-27

**Authors:** Agnieszka Wojnarowska, Dorota Kobylinska, Karol Lewczuk

**Affiliations:** ^1^Faculty of Psychology, University of Warsaw, Warsaw, Poland; ^2^Institute of Psychology, Cardinal Stefan Wyszynski University, Warsaw, Poland

**Keywords:** acceptance, emotion regulation strategies, instructing acceptance, experiments on emotion regulation, emotion regulation

The concept of acceptance, understood as a self-regulation strategy based on an open and welcoming attitude toward one's own emotions, thoughts, or external events (Williams and Lynn, 2010)^1^, is present in various domains of psychological research and practice. Previous studies have brought important knowledge on the nature of this strategy, but very significant gaps in the knowledge still exist.

The most important issues are as follows: (1) conceptual difficulties regarding acceptance as an emotion regulation strategy; (2) lack of coherence in operationalizations of acceptance in various research; and (3) acceptance not being recognized as a distinct emotion regulation strategy in the most influential emotion regulation models. In the present paper we highlight and discuss these issues in more detail and—based on this discussion, postulate directions for future experimental research on acceptance.

The popularity of research on acceptance has grown steadily since the 1990s, when acceptance-based therapeutic approaches started to develop more rapidly. For example, the role of acceptance was underlined in the Acceptance and Commitment Therapy (ACT; Hayes et al., 1999), as well as other approaches.

Many different forms of acceptance-based programs were developed and tested for effectiveness. They were shown to be successful in stress and pain reduction as well as decreasing anxiety and depression symptoms (Segal et al., 2002; Hayes et al., 2006; Veehof et al., 2011; Twohig and Levin, 2017; Feliu-Soler et al., 2018). Other studies showed the role of mindfulness and acceptance for the severity of psychotic symptoms (Cramer et al., 2016; Jansen et al., 2019), eating disorders (Prefit et al., 2019), compulsive sexual behavior (Lew-Starowicz et al., 2019), addictive behaviors (Bowen et al., 2011), suicidal ideation and self-harm (Tighe et al., 2018) as well as other psychopathological symptom clusters (Aldao et al., 2010).

The definitional goal of acceptance as an emotion regulation strategy is not to change the experienced emotions, but to receive them without control attempts (Hayes, 2004; Kohl et al., 2012). Thus, acceptance is quite distinct from other frequently studied ways of regulating emotion (e.g., suppression, most forms of cognitive reappraisal, rumination) that are most often based on some form of active modification of emotional state in terms of quality, strength, length, or frequency of emotion (Gross, 2015). Despite these differences, acceptance is present in psychological research on emotion regulation and is often compared with other regulatory strategies (e.g., Liverant et al., 2008; Aldao et al., 2010; Naragon-Gainey et al., 2017; Southward et al., 2019). However, despite the presence of acceptance in the work of both psychological practitioners and theoreticians, we stumble upon significant difficulties when trying to find acceptance in broader theoretical models of emotion regulation. ACT has its theoretical roots in the theory of Relational Frames (Barnes-Holmes et al., 2001). However, many techniques designed to support emotional acceptance that are applied in therapeutic practice itself are not very close to the theoretical model. What is crucial is that acceptance is not explicitly present within the most influential model of emotion regulation, i.e., Gross's process model (Gross, 1998), although it is present in others (Gratz and Roemer, 2004; Berking et al., 2008). According to the conception put forward by Gratz and Roemer (2004), (a) non-acceptance of emotion and (b) lack of emotional awareness and (c) clarity are three out of six of the most important areas of difficulties in emotion regulation.

Some authors suggest that within Gross's model, acceptance should be classified within the attentional deployment strategies' family (e.g., Slutsky et al., 2017), while others see it as a form of reappraisal (e.g., Webb et al., 2012), depending on whether they focus on either acceptance as influencing attention or acceptance as a way of understanding the whole emotional experience. These two approaches to acceptance-based strategies hint at another important issue visible in the acceptance literature: lack of conceptual clarity and stark differences in operationalizations of this emotion regulation strategy. This leads to difficulties in integrating the results of research on acceptance and possibly to high variability in the results of research on acceptance effectiveness. This point will be elaborated on in following sections. Previous research contains attempts at placing acceptance-related strategies within other classes of regulation emotion strategies and studying the underlying factor structure (see e.g., Naragon-Gainey et al., 2017), although more research on this front is needed to provide us with reliable and parsimonious solutions.

According to a meta-analysis by Webb et al. (2012), acceptance-like strategies are—on average—effective (*d* = 0.30, the effect sizes are reported in terms of Cohen's d, Cohen, 1988)—their effectiveness is, moreover, higher than the effectiveness of suppression (*d* = 0.03), similar to distraction (*d* = 0.31), but lower than some other forms of reappraisal, like perspective taking (*d* = 0.61). However, as mentioned above, there is a very high variability between the results of particular studies (for a meta-analysis, see Kohl et al., 2012). Some results indicate that acceptance is more effective, and some indicate that it is less effective when compared to other strategies, like reappraisal (Kohl et al., 2012; Webb et al., 2012; Smoski et al., 2015). One recent study showed for example that acceptance can be ineffective on the level of emotional experience, while still successfully downregulating psychophysiological responses (Boehme et al., 2019), although not all studies led to similar findings (Kohl et al., 2012). In our view, these differences can be partially ascribed to the fact that, in various studies, qualitatively different self-regulatory strategies are activated under one joint label, *acceptance*. When operationalizing acceptance, researchers most often refer to ACT theory, but available studies differ in, for example, the number of ACT components that a particular self-regulation instruction addresses. ACT consists of 6 components: (1) willingness to take in emotions, (2) being present (mindfulness), (3) cognitive defusion, (4) self as a context, and (5) concentration on values and (6) commitment (Hayes et al., 1999).

To illustrate the issue of differing operationalizations of acceptance in experiments, we can use a representative example of an instruction:

The instruction refers to two components distinguished in ACT: (1) willingness (Table 1), which is readiness and openness to fully experiencing emotion (Hayes et al., 2006) and (2) being present, which is related to concentration on the present moment (Hayes et al., 2006). Instructions based on willingness stress the lack of necessity to control, modify, or intervene in emotional processes. While when instructions refer to being present and mindfulness, participants are asked to keep their attention focused on emotions, thoughts and feelings they are experiencing at a particular moment (e.g., Segal et al., 2002; Singer and Dobson, 2007). Recent work brought the first evidence that the two described processes, willingness, and mindfulness, can have differential consequences for emotion regulation outcomes (Lindsay and Creswell, 2016). When these two components are applied in conjunction, they support effective emotion regulation; however, when mindfulness is applied without acceptance, it leads to the strengthening, and not reducing, negative emotional reactions, such as anxiety and stress (Barnes and Lynn, 2010; Desrosiers et al., 2014).

**Table 1:** Example of an instruction and its analysis.

**Reference**	**Instruction for participants**	**What participants are instructed to**
Dunn et al. (2009), p. 764	“It is very important for the experiment that when you watch the film you try and accept any emotional responses to it you are having. Immerse yourself in the film, allowing yourself to internally experience and externally express any emotions it produces. Rather than trying to control your reaction imagine your emotion is like a cloud passing in the sky—a natural phenomena that comes and goes regardless of any attempts you make to influence it. (g) Let the feelings wash over you, (h) being aware of how they make you think, feel and react. Just (i) observe all the different aspects of how you are feeling in response to the film, (j) rather than judging whether the emotion is “good” or “bad” or “wanted” or “unwanted.”	(a) Accept (b) Immerse themselves (c) Experience internally (d) Express externally (e) Don't control (f) Imagine their emotion is like a cloud passing in the sky (g) Let the feelings wash over them (h) Be aware (i) Observe (j) Don't judge

Moreover, in some of the instructions, aimed at activating acceptance in experiments, an internal monolog is encouraged and examples of self-talk are given (Singer and Dobson, 2007; Matthies et al., 2014). Others invite participants to change their attitude toward emotion and thoughts in a non-discursive manner (discursive thought is not the main tool of emotion regulation in such cases) (e.g., Wolgast et al., 2011). The distinction between discursive and non-discursive strategies seems very interesting, but it has also been completely overlooked—as of now, we do not have any studies systematically comparing these two ways of instructing emotion regulation strategies.

Another type of instruction is designed to activate the next ACT component—the process of cognitive defusion—the ability to separate from one's thoughts and emotions and allow them to come and go. Defusion's primary function is to change the status of regulated emotions instead of changing their length or strength directly. Defusion decreases the believability of private experiences, thereby decreasing reliance on and attachment to one's own emotions (Hayes et al., 2006). In some of the previous studies, cognitive defusion instruction consisted of the rapid vocal repetition of one word (e.g., “milk”), which also prevents discursive thinking and suggests that it could also be effective in dealing with self-referential negative thought (Masuda et al., 2010). In another study, participants were encouraged to *disconnect their thoughts from their feelings* and to *notice their thoughts and feelings, but not allow them to control their behavior* (Keogh et al., 2005, p. 593). In still another one, participants were taught to *step back from cravings* and *see themselves having them* (Forman et al., 2007, p. 2377). It is worth noting that the kind of diffusion that is based on stepping back from emotional experience (distancing) has been studied within another emotion management tradition: memory research (as self-distancing Ayduk and Kross, 2010; Kross and Ayduk, 2017) and within Gross's process model (as distancing; Ochsner et al., 2004). However, in research related to Gross's process model, distancing is conceptually different from acceptance as a strategy, which is another sign of the lack of conceptual clarity within the field of emotion regulation research (Ochsner et al., 2004; Webb et al., 2012).

There is also research in which another ACT process is invoked—concentration on values. In instructions that operationalize this process, participants are encouraged to be open to their experiences, while simultaneously concentrating on behavior change in valued directions (Levitt et al., 2004). This ACT component has a behavioral and evaluative element (focusing on elements of experience that are deemed to be important), which is not the case for other genres of acceptance. While this subject needs more research, we argue that this component can be viewed as assisting, but not a core element of acceptance strategy.

To sum up, even a single look at different ways of instructing acceptance as emotion regulation strategy shows that completely different processes may be activated by these different instructions, as being present, willingness, diffusion, and concentration on values are starkly different mental processes (see Kohl et al., 2012). Treating them as the same process, or mixing them within one instruction without sufficient care about their specific effects, is in our view not a useful approach and a missed opportunity to learn more about the nature of acceptance as a regulatory strategy. We postulate that to be able to more reliably study acceptance in experimental research on emotion regulation, researchers should: (1) demonstrate if various acceptance components are different or not (2) if they are—researchers should investigate the effectiveness of various acceptance components separately, as well as (3) systematically study combinations of acceptance components in a controlled way. This would give us better understanding of the mechanisms that underlie the effectiveness of acceptance.

Lastly, we wanted to highlight some additional, methodological factors that could possibly influence the results of experimental studies centered on acceptance as emotion regulation strategy. Aside from the conceptual differences in the operationalizations described above, there are also other important discrepancies between studies that are strictly methodological in nature. The acceptance instructions have different lengths, level of detail and level of complexity. Some of them include examples, metaphors or additional exercises (Gutiérrez et al., 2004; Roche et al., 2007; McMullen et al., 2008), while others do not. Sometimes, benefits of using acceptance are described in the instruction itself (so the effectiveness of the strategy is effectively primed; Levitt et al., 2004). In some studies, participants just read the instruction (Dunn et al., 2009), whereas in others they complete short practice exercises (Eifert and Heffner, 2003) or even participate in a longer training (Hayes et al., 1999). Available results suggest that acceptance requires longer training to be effective than other, simpler strategies (Baer et al., 2012; Desbordes et al., 2015), and only the participants who knew the strategy before were able to use it effectively after a short training session, to deal with the experience of pain (Blacker et al., 2012). It seems that the discussed methodological differences can have significant importance for the outcomes of the research, as they lead to differential consequences on the cognitive (e.g., the level of comprehension and memorization of instruction) as well as motivational level (e.g., willingness to apply the instruction)—however, in most research, these factors are not controlled. Additionally, recent research showed that effectiveness of emotion regulation strategies can be dependent on the specific emotion that is targeted in the regulation episode, which should be further explored in future studies (Southward et al., 2019). Also, it appears that, under particular circumstances, the use of acceptance can be greater among older adults (Allen and Windsor, 2019), so the age of participants should be systematically examined in research.

To conclude, in light of the current state of research, as well as the discussed deficiencies in knowledge on acceptance as an emotion regulation strategy, we postulate a more thorough and systematic approach to conceptualizing and studying this strategy, taking into account various and distinct acceptance components and other methodological factors that can contribute to acceptance effectiveness. We encourage researchers to pay more attention to: (1) placing acceptance in the existing emotion regulation conceptualizations, (2) controlling different components of acceptance that are activated through instructions, and (3) the issue of training (and its length) of the strategy in the course of a study.

## Author Contributions

AW came with the idea for the paper and prepared the outline. AW and DK prepared the literature review. All three authors participated in preparing the first and second draft of the manuscript.

### Conflict of Interest

The authors declare that the research was conducted in the absence of any commercial or financial relationships that could be construed as a potential conflict of interest.

## Footnotes

^1^It is noteworthy that this definition of acceptance includes two forms of acceptance: of the situation and of emotions. The articles on acceptance rarely contain clear definition of this strategy, sometimes they even don't contain definition at all (Hayes, 2004; Aldao et al., 2010; Kohl et al., 2012). Research usually concerns only the first form (acceptance of emotions).
